# Aortic dissection: Indecision and delays are the parents of physiological failure

**DOI:** 10.1113/EP091964

**Published:** 2024-06-04

**Authors:** Damian M. Bailey, Mohamad Bashir, Ian M. Williams

**Affiliations:** ^1^ Neurovascular Research Laboratory, Faculty of Life Sciences and Education University of South Wales Pontypridd UK; ^2^ Vascular and Endovascular Surgery Health & Education Improvement Wales Wales UK; ^3^ Department of Vascular Surgery University Hospital of Wales, Health Park Cardiff Wales UK

A recent publication in a national newspaper in the UK reported the sudden and unexpected death of an apparently fit and well 50‐year‐old female returning from holiday after complaining of chest pain as the plane began its descent to land (Raemason, 2024). The cause of death was an acute aortic dissection (AD), an infrequent yet potentially catastrophic disorder affecting the abdominal and thoracic aortic segments. The coroner's inquest concluded that the death was probably avoidable due to significant delays of up to 16 h prior to operative intervention being attempted. In this editorial, we briefly examine the integrative physiology and anatomical–surgical challenges and controversies associated with this emergent and potentially deadly medical condition.

A tear in the aortic intima is the cause of AD, which results in a blood column penetrating the medial layer of the aorta causing a ‘hydraulic endarterectomy’, creating a septum of medial and intimal aortic tissue that separates the aorta's true and false lumens (Figure 1a). AD has an estimated incidence of between 4.5 and 7 per 100,000 (Booth, 2023) and if left untreated, mortality can be as high as 90% after 3 months, with acute mortality estimated between 1% and 2% per hour after the onset of symptoms (Kouchoukos & Dougenis, 1997; Tsai et al., 2005).

**FIGURE 1:**
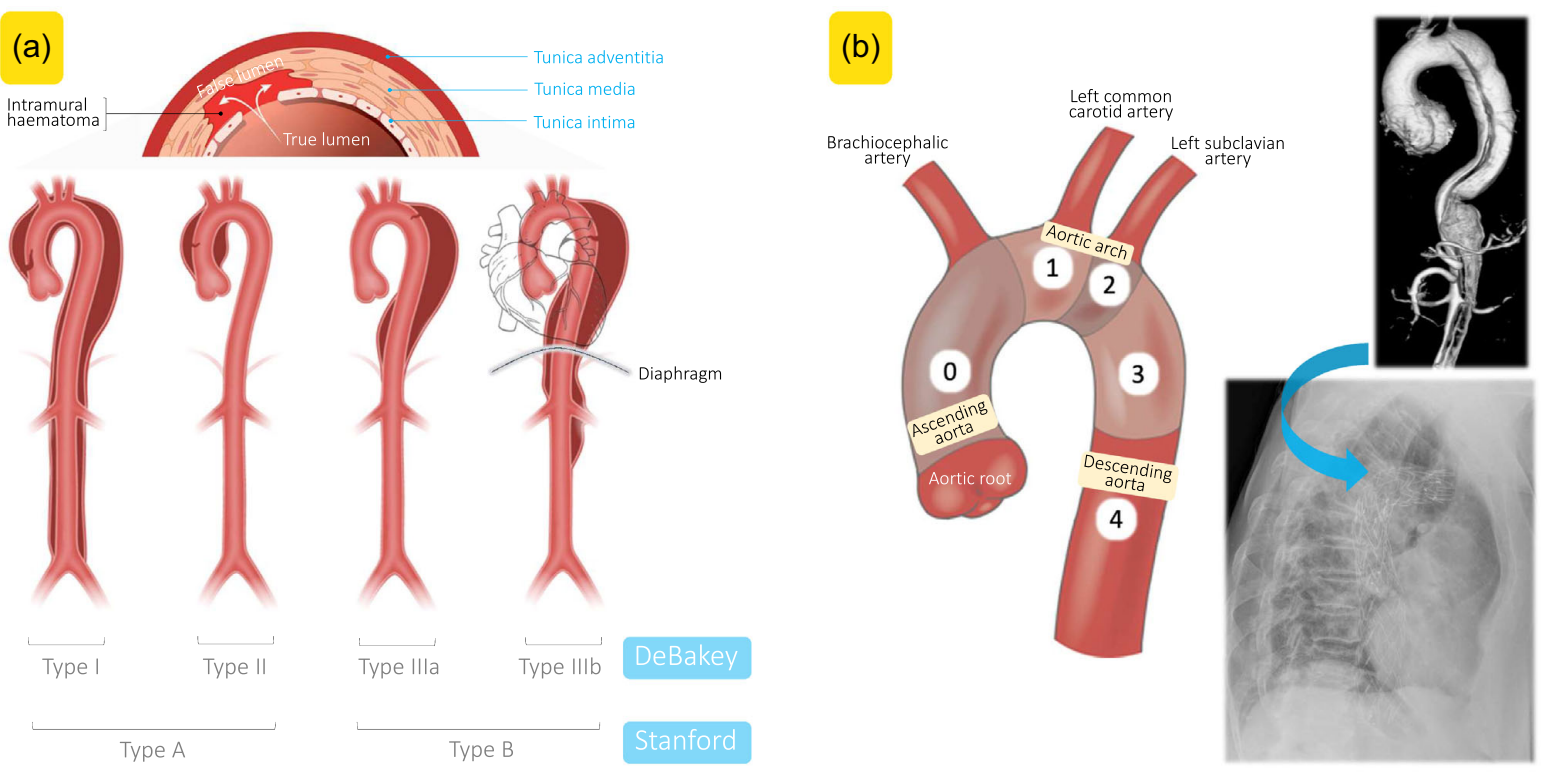
DeBakey and Stanford aortic dissection classification systems (a) and zones for the ascending aorta and arch (b). (a) In aortic dissection, a tear in the intima layer results in blood moving into the media, developing into an intimal flap and dividing the original vessel into a true and false lumen. The DeBakey system classifies lesions on the basis of the location of the initiating intimal tear and the pattern of extension. DeBakey Type I and II lesions involve the ascending aorta, with Type II involvement limited to the ascending aorta. DeBakey Type III lesions are subdivided into type IIIa if the lesion involves only the descending aorta and extension is limited to above the diaphragm, and Type III b if the lesion involves only the descending aorta but extends to below the diaphragm. The Stanford system classifies lesions as Type A if the ascending aorta is involved, anywhere from the root to the proximal origin of the brachiocephalic artery. All other lesions are considered Stanford Type B. Created with BioRender.com. (b) Note multiplanar reformation image by magnetic resonance angiography of Type B aortic dissection highlighting morphologic details in 3D and corresponding repair with stent graft (right). Images authors’ own copies.

Clinical diagnosis of AD is challenging and remains controversial, complicated by its modest prevalence, lack of sensitive–specific biomarkers and highly variable clinical course with a relatively high number of atypical presentations (Banceu et al., 2024). In addition to established risk factors, with severe chronic hypertension the most common, sudden onset chest/aortic pain is a common symptom that is typically sharp, strong and tearing, often radiating towards the destination of the lesion's advancement in accordance with the implicated aortic branches (Erbel et al., 2014). A 12‐lead electrocardiogram, chest radiography and diagnostic biomarkers (e.g., systemic concentrations of cardiac troponin and D‐dimer) help exclude differential diagnoses of pulmonary embolism or acute coronary syndrome. In the emergency room, a computed tomography aortogram is the favoured imaging diagnostic modality of choice in light of widespread accessibility and comprehensive anatomical evaluation of the aorta and branch vessels (Vardhanabhuti et al., 2016). Post‐contrast computed tomography (CT) of the whole (thoracic and abdominal) aorta is recommended in high‐risk patients including those with known aortic disease to assess the full extent of dissection and involvement of branch vessels (Callaway et al., 2024).

AD has traditionally been classified as either Type A or B with a prevalence of 70% and 30%, respectively. Type B aortic dissection (TBAD) is recognised as involving the descending thoracic or abdominal aorta whilst Type A (TAAD) involves the ascending aorta. The original DeBakey classification in 1965 describes the dissection according to the origin of the tear within the aortic wall (DeBakey et al., 1965). A Type I classification involves the ascending aorta, aortic arch and descending aorta whilst a Type II is confined to the ascending aorta only. A Type III dissection is limited to the descending thoracic aorta and has been divided into A and B depending on the distal extent of the flap (Figure 1a).

The Stanford classification was published in 1970 and specifically focused on whether the ascending thoracic aorta was involved or not but failed to address the extent of the dissection (Daily et al., 1970). Stanford A correlates with DeBakey Types I and II and invariably requires urgent intervention. It soon became apparent there was a subset of AD originating in or involving the aortic arch that did not fit within the existing definitions of either the Stanford or the DeBakey classification. These either have a primary entry tear in the aortic arch or are a retrograde dissection extending proximally into the arch but sparing the ascending aorta. These have been classified as a non‐A, non‐B dissection with an estimated prevalence of 3%–16% (Sievers et al., 2020). Hence this represents a significant number of patients presenting with AD.

The classification for reporting TBAD was further refined in 2020 (Lombardi et al., 2020). The authors stated that an AD was a Type B if the TBAD entry tear occurred in zone 1 or distally (within the arch) whilst a TAAD was confined to zone 0 (Figure 1b). This was developed as an easier way of describing the dissection for reporting purposes. As well as early recognition of AD, it is essential in order that the appropriate treatment can be instigated to ascertain the type of dissection. In the case of TAAD surgical intervention is invariably the preferred treatment. For TBAD, after initial stabilisation with pain control and antihypertensives, an endovascular option rather than open surgery is now invariably considered at an aortic specialist centre. Non‐A and non‐B AD are more complicated to treat as (surgically) adequate proximal ‘landing zones’ may not be available and vital arteries to the cerebral circulation and upper limbs need to be perfused (Figure 1b).

The UK has suffered from a lack of a network management of AD, which is concerning given that AD is a time critical diagnosis and a high index of suspicion is required at receiving accident and emergency units when a patient presents with the condition. The medical care for TBAD has been under the auspices of surgeons, interventionalists and cardiologists, but due to varying symptoms at presentation it can be misdiagnosed leading to significant and life‐endangering delays (Bashir et al., 2023; Jubouri et al., 2023). A family history of aortic disease, known aortic valve disease, recent aortic procedure, and thoracic aortic dilatation or aneurysm with an aortopathy means AD must be excluded as a cause of symptoms.

In March 2021, The Acute Aortic Dissection Toolkit was proposed to provide management plans for the initial treatment once the diagnosis is made (NHS, 2024a). In addition, a recent meeting in Oxford (UK) proposed the establishment of supraregional centres in those presenting with AD in South England (NHS, 2024b). It was further suggested that non‐A and non‐B AD should be treated as if they were a TAAD with the appropriate urgent referral to a regional cardiac unit.

Furthermore, in an attempt to reduce delays and accelerate diagnosis, an AD Detection Risk Score was published (Rogers et al., 2011). Three categories were considered: associated conditions (aortopathies, known aortic valve disease, etc.), pain and clinical findings. A score of 0 or 1 was awarded per category with a score of 0 deemed low risk for a dissection. A score of 1 is intermediate where you would consider a CT scan whilst a score of 2–3 is high risk for a dissection. Anti‐impulse therapy is vital and must be commenced as soon as possible and continued even if a hospital transfer is performed.

Accurate classification of AD is essential as this dictates the treatment plan followed and has been overdue given that the original DeBakey and Stanford systems were published when imaging modalities, including CT, were not available and there was no option for endovascular surgery. Furthermore, TAAD invariably requires urgent surgical intervention whilst TBAD is initially treated conservatively, with a subsequent stent graft should certain high‐risk radiological features or clinical symptoms be present. Aortic procedures have progressed also with techniques such as the elephant trunk, hybrid arch repair and extra‐anatomical bypass grafting performed enabling proximal landing zones for a stent graft into zone 0. This is particularly the case for treating complex aortic arch (non‐A non‐B) dissections.

Predictions have suggested that the UK population aged over 75 years will increase from 8% to 15% by 2050 (Howard et al., 2013). Thus, the incidence of AD could potentially double in this time period with the majority being TAAD (which also has the highest mortality rates). A treatable major risk factor for the development of AD is uncontrolled hypertension, and an aggressive approach to this may reduce future numbers of AD occurring and reduce mortality rates. With the development of regional network services treating AD in the future, prospective registries can enable the natural history of the condition to be followed and interrogated.

Guidelines for urgent imaging to establish a diagnosis and instigate immediate treatment will undoubtedly reduce mortality. The management of acute chest pain in emergency units has traditionally been to exclude a myocardial infarction or pulmonary embolism. However, the immediate use of thrombolytics to disperse a clot, including pre‐emptive anticoagulants and platelet aggregation inhibitors, may have a detrimental effect on patient care particularly if AD is diagnosed and operative treatment may need to be delayed. Coronary artery angioplasty is now the preferred treatment over clot‐dissolving therapy. Primary angioplasty for acute myocardial infarction should be performed as soon as possible for the best results. In case of a technical delay, thrombolytic therapy may be used as an initial treatment to ‘buy’ time. However, thrombolytic therapy is not suitable for all types of myocardial infarction, and is limited to ST segment elevation. This is because non‐ST elevation myocardial infarction typically associates with a firmer platelet‐rich white thrombus and is unlikely to have a soft red thrombus that can be dissolved with thrombolytic therapy. However, fondaparinux sodium, a synthetic pentasaccharide that selectively binds to antithrombin III catalysing the inhibition of factor Xa, is used in acute coronary syndrome. Hence, the use of antiplatelets and fondaparinux potentiate post‐operative bleeding following TAAD repair.

With the necessary education and awareness in place, AD should be considered and rapidly excluded as a cause of the presenting chest pain. Accurate and consistent AD classification is required to reduce delays in order for the correct treatment plan to be followed and, ultimately, mortality rates reduced. The sporadic albeit important reporting in the national press of deaths from AD brings momentary attention to a complicated disease which has considerable mortality and lifelong morbidity. Reporting such as this can only help with the introduction of the word ‘dissection’ into normal differential diagnosis parlance with the ultimate goal of saving lives.

## AUTHOR CONTRIBUTIONS

Damian M. Bailey conceived the idea and with Ian M. Williams, wrote the first draft of the manuscript. Damian M. Bailey, Mohamad Bashir and Ian M. Williams edited and revised the manuscript. All authors have read and approved the final version of this manuscript and agree to be accountable for all aspects of the work in ensuring that questions related to the accuracy or integrity of any part of the work are appropriately investigated and resolved. All persons designated as authors qualify for authorship, and all those who qualify for authorship are listed.

## CONFLICT OF INTEREST

D.M.B. is Editor‐in‐Chief of *Experimental Physiology*, Chair of the Life Sciences Working Group, member of the Human Spaceflight and Exploration Science Advisory Committee to the European Space Agency and member of the Space Exploration Advisory Committee to the UK Space Agency. He is also affiliated to Bexorg, Inc. (USA) focused on the technological development of novel biomarkers of cerebral bioenergetic function and structural damage in humans.

## FUNDING INFORMATION

This work was funded by Royal Society Wolfson Research Fellowship. Grant No. WM170007.
